# The longitudinal impact of social media use on adolescent mental health in the UK

**DOI:** 10.1093/eurpub/ckac129.054

**Published:** 2022-10-25

**Authors:** R Plackett, J Sheringham, J Dykxhoorn

**Affiliations:** 1 Primary Care & Population Healt, University College London, London, UK; 2 Department of Applied Health Research, University College London, London, UK

## Abstract

**Background:**

Cross-sectional studies have suggested a relationship between social media use and depression and anxiety in young people. We examined the longitudinal relationship between social media use and young people's mental health and the role of self-esteem and social connectedness as potential mediators.

**Methods:**

The sample comprised 3,228 young people from the UK Longitudinal Household Study (waves 1-10). Mental health at age 14 or 15 was measured by the SDQ Total Difficulties score. The number of hours spent on social media was measured at age 12 or 13. Self-esteem at age 13 or 14 was measured via eight questions and social connectedness was measured by two questions. Multilevel linear regression models explored whether social media use at age 12 or 13 predicted mental health at age 14 or 15. Path analysis with structural equation modelling investigated the mediation pathways.

**Results:**

In unadjusted analysis, for those who spent 7 or more hours on social media vs none, their mental health problems trended upwards by 3.87 (95% CI, 0.71-7.03) but this relationship was attenuated after including covariates. In unadjusted path analysis, more social media use was associated with lower self-esteem (b=-0.10, p < 0.05), which in turn was associated with more mental health problems (b=-6.80, p < 0.001). The indirect effect (b = 0.70, p < 0.05) showed that 68% of the effect of social media use on mental health two years later was mediated by self-esteem. This relationship was attenuated after adjusting for covariates and in imputed data, and social connectedness was not associated.

**Conclusions:**

This study shows the importance of longitudinal evidence, as we found there was little evidence to suggest a causal relationship between social media use and mental health issues two years later. Interventions that address social media use alone may not improve young people's mental health but those that consider factors like self-esteem may be more effective.

**Key messages:**

• Longitudinal data suggests there is limited evidence that high social media use causes poorer mental health in adolescents despite indications from cross-sectional analyses.

• Policy makers should consider that targeting social media use alone is unlikely to prevent poor adolescent mental health and factors like self-esteem may be more important prevention targets.

